# Interleukin-6 in Allogeneic Stem Cell Transplantation: Its Possible Importance for Immunoregulation and As a Therapeutic Target

**DOI:** 10.3389/fimmu.2017.00667

**Published:** 2017-06-08

**Authors:** Tor Henrik Anderson Tvedt, Elisabeth Ersvaer, Anders Aune Tveita, Øystein Bruserud

**Affiliations:** ^1^Department of Clinical Science, Section for Hematology, University of Bergen, Bergen, Norway; ^2^Department of Medicine, Haukeland University Hospital, Bergen, Norway; ^3^Institute of Biomedical Laboratory Sciences and Chemical Engineering, Western Norway University of Applied Sciences (HVL), Bergen, Norway; ^4^Department of Immunology and Transfusion Medicine, Oslo University Hospital, Oslo, Norway

**Keywords:** interleukin-6, cytokine receptor gp130, allogeneic stem cell transplantation, graft-versus-host disease, Janus kinases

## Abstract

Allogeneic stem cell transplantation is associated with a high risk of treatment-related mortality mainly caused by infections and graft-versus-host disease (GVHD). GVHD is characterized by severe immune dysregulation and impaired regeneration of different tissues, i.e., epithelial barriers and the liver. The balance between pro- and anti-inflammatory cytokine influences the risk of GVHD. Interleukin-6 (IL-6) is a cytokine that previously has been associated with pro-inflammatory effects. However, more recent evidence from various autoimmune diseases (e.g., inflammatory bowel disease, rheumatoid arthritis) has shown that the IL-6 activity is more complex with important effects also on tissue homeostasis, regeneration, and metabolism. This review summarizes the current understanding of how pro-inflammatory IL-6 effects exerted during the peritransplant period shapes T-cell polarization with enhancement of Th17 differentiation and suppression of regulatory T cells, and in addition we also review and discuss the results from trials exploring non-selective IL-6 inhibition in prophylaxis and treatment of GVHD. Emerging evidence suggests that the molecular strategy for targeting of IL-6-initiated intracellular signaling is important for the effect on GVHD. It will therefore be important to further characterize the role of IL-6 in the pathogenesis of GVHD to clarify whether combined IL-6 inhibition of both trans- (i.e., binding of the soluble IL-6/IL-6 receptor complex to cell surface gp130) and cis-signaling (i.e., IL-6 ligation of the IL-6 receptor/gp130 complex) or selective inhibition of trans-signaling should be tried in the prophylaxis and/or treatment of GVHD in allotransplant patients.

## Introduction

Allogeneic stem cell transplantation (ASCT) is a potentially curative therapeutic modality used primarily in the treatment of hematological malignancies and bone marrow failure syndromes (1, 2). Despite better supportive care and increased understanding of the underlying pathophysiology processes, the majority of patients will experience either acute or chronic graft-versus-host disease (GVHD) (3).

Graft-versus-host disease is a complex biological process, which includes severe immune dysregulation with sustained inflammation (4). Several studies have identified interleukin-6 (IL-6) and as an important factor in the pathophysiology of GVHD (5, 6). IL-6 is an important cytokine involved in both acute and chronic inflammation, and it plays a major role in several autoimmune disorders (7–9). It also mediates strong anti-inflammatory effects important for immunoregulation, metabolic control, and tissue regeneration (10). During the last 15 years the pleiotropic effects of IL-6 in different organ systems have been reveled, and anti-IL-6 treatment has been approved for autoimmune and neoplastic disorders (11). With increasing insight into the complex signaling events induced by IL-6, more specific blockade of the anti-inflammatory functions of IL-6 is under development.

## ASCT and GVHD

In current ASCT protocols an initial conditioning treatment with chemotherapy and/or total body irradiation is followed by infusion of a stem cell graft derived from either umbilical cord blood, bone marrow, or peripheral blood of a healthy donor. Severe GVHD occurs in 20–30% of adult allotransplant recipients and is an important cause of transplant-related mortality (12, 13). GVHD is classified as either acute or chronic GVHD based on clinical characteristics. According to the clinical definitions, classic acute GVHD (aGVHD) develops within 100 days posttransplant with distinctive clinical features in the skin, gastrointestinal (GI) tract, or liver. However, manifestations of aGVHD can reoccur, persist, or present after day 100 posttransplant and is then classified as recurrent, persistent, or late onset aGVHD. Classical chronic GVHD is not limited to specific organ system and can present at any time after transplantation. The diagnosis of chronic GVHD is based on consensus criteria for each organ system involved. While some clinical features are diagnostic (e.g., bronchiolitis obliterans and sclerotic features of the skin), other manifestations require additional clinical and/or histological criteria to be fulfilled. Some patients may also present as an overlap syndrome with clinical features of both acute and chronic GVHD.

Scoring systems have been developed to grade the severity of aGVHD based on the involvement of skin, GI-tract, and liver; the Glucksberg scale (grading aGVHD from I to IV) or the International Bone Marrow Transplant Registry (grading from A to D) are most widely used (14). First-line treatment for grade II–IV aGVHD is high-dose steroids (15), and a complete response is observed in 20–40% of patients (16, 17). There is no consensus regarding the preferred therapy for steroid-refractory aGVHD; commonly utilized options include TNFα blockade, anti-IL-6 antibodies, mTOR inhibitors, mycophenolate mofetil, and extracorporeal photopheresis (18). Despite several therapeutic alternatives, patients with grade IV steroid-refractory aGVHD have a dismal long-term prognosis (15).

**Figure 1:**
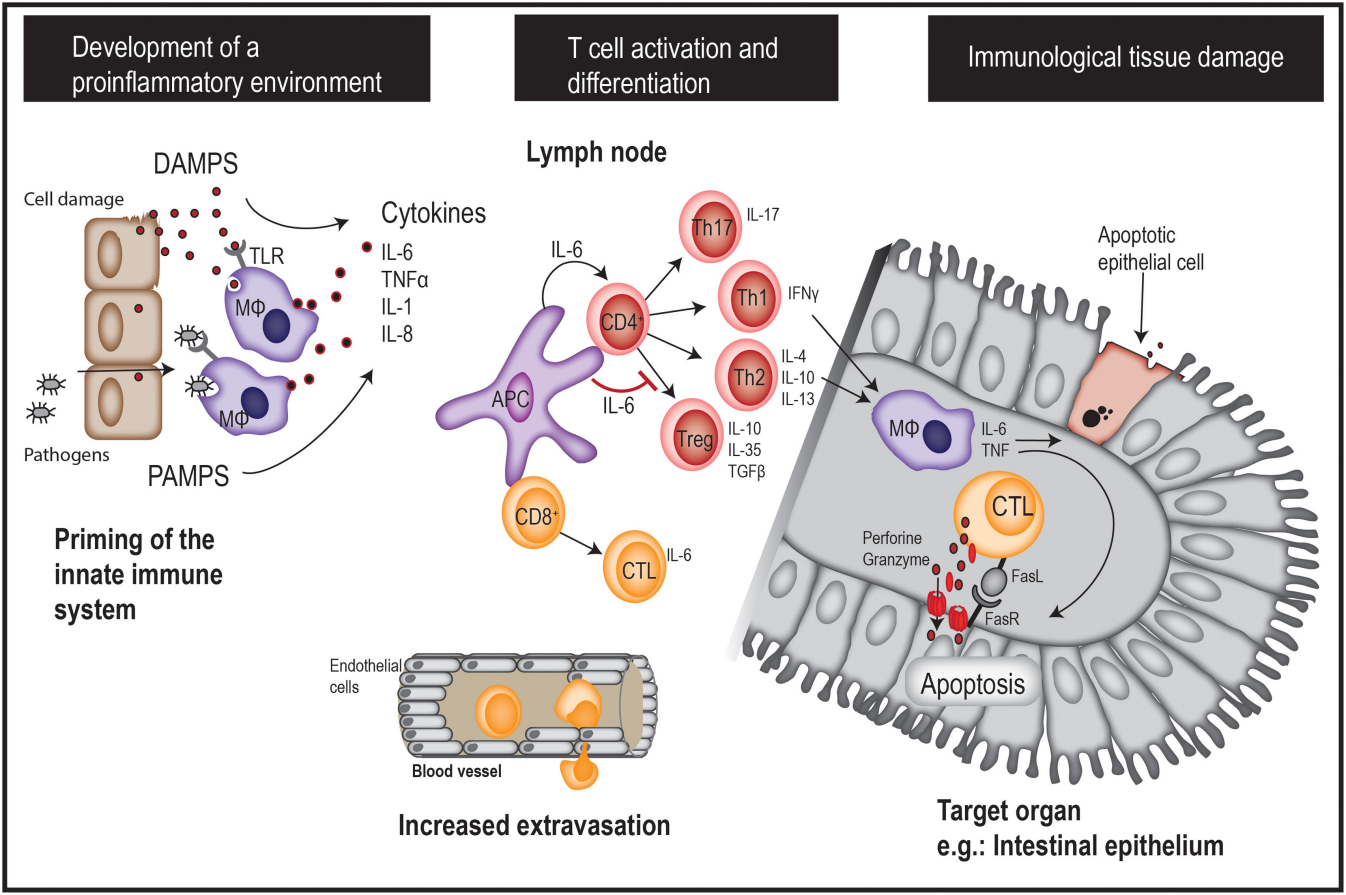
A brief overview of acute GVHD pathogenesis. GVHD, graft-versus-host disease; DAMPS, danger-associated molecular patterns; PAMPS, pathogen-associated molecular patterns; APC, antigen-presenting cell; MΦ: macrophage.

Donor T cells play major roles in the development of aGVHD, but multiple factors contribute to the overall risk of severe aGVHD. The development of aGVHD is believed to be a three-step process each involving different subsets of immune cells (Figure 1) (19, 20). The first step is characterized by activation of the innate immune system; this results in a state of systemic inflammation prior to the introduction of donor T cells. The chemotherapy and/or radiation used in the conditioning therapy invoke tissue damage with release of pro-inflammatory cytokines, e.g., IL-6, TNFα, IFN-γ, and IL-1. Damage to the GI barrier allows increased translocation of microorganisms from the gut microbiome, with subsequent increased levels of circulating lipopolysaccharide as well as molecules with pathogen- and damage-associated molecular patterns (PAMPs/DAMPs) (21). As a consequence, antigen-presenting cells (APC) secrete pro-inflammatory cytokines and present host as well as pathogen peptides on their major histocompatibility complex molecules. A reduction of pretransplant inflammation (e.g., through the use of reduced intensity conditioning, reduction of gut microbiome levels) and the presence of genetic factors associated with reduced inflammatory responses are associated with a reduced risk of GVHD and treatment-related mortality (22–24). Several other factors also influence the pretransplant pro-inflammatory state, e.g., pro-inflammatory metabolites, soluble adhesion molecules, and factors associated with altered endothelial cell functions with increased vascular permeability (6, 25–27).

The second step is characterized by alloreactive T-cell activation, proliferation, and differentiation as a response to the presentation of host antigens by host APC in a proinflammatory context. Stimulation of specific T helper cell subsets is thought to be important for the initiation of later cytotoxic T-cell-mediated tissue damage (19, 20). Th1 cells release interferon-γ (IFN-γ) at high levels and express the transcription factors STAT4 and STAT1/T-bet (28, 29). This Th1 polarization seems to be important for the development of aGVHD, especially in the GI tract (30). Additionally, Th2 cells characterized by expression of the transcription factor GATA-3 and secretion of anti-inflammatory cytokines IL-4, IL-10, and IL-13 (31) may also play a role in GVHD pathogenesis. The available studies of Th2 cells in GVHD have given conflicting results, but some studies have described an association between Th2 differentiation and pulmonary as well as skin involvement in aGVHD (30, 32, 33). Th17 cells are characterized by the expression of the transcription factor RORγt and IL-17 secretion (34), and IL-17-secreting T cells are thought to be important for aGVHD severity (35) as well as for early transplant-related severe lung injury (36). Finally, T regulatory cells are a specialized Th subset characterized by expression of the transcription factor FOXP3 and high IL-2 receptor (CD25) expression. These cells play an important regulatory role by actively suppressing immune responses through their release of the anti-inflammatory cytokines IL-10, TGF-β, and IL-35 (37), and through direct interaction with other T cell subsets. Tregs are suppressed during GVHD, and resolution of GVHD is associated with restoration of the Treg function (38). Of particular interest, given the current topic of discussion, a majority of early posttransplant circulating TCRαβ^+^ CD4^+^ and CD8^+^ T cells release IL-6 at relatively high levels together with classical proinflammatory cytokines such as IFN-γ and TNFα (39).

The third step of GVHD development is characterized by local action of cytotoxic CD8^+^ T cells triggered by the local release of chemokines during the two first steps; these cells mediate direct cytotoxic effects upon target cell recognition, including secretion of perforin/granzyme and FAS-ligand. Other T-cell subsets contribute to the organ-specific manifestations of GVHD through polarization of macrophages toward a pro-inflammatory (M1) phenotype that further increase tissue damage through the release of oxidants and pro-inflammatory cytokines including TNF-α and IL-6 (40, 41).

## IL-6 and Its Receptors—Classical Signaling and Trans-Signaling

### The Structure and Release of IL-6

Interleukin-6 is a glycosylated protein with a molecular weight of 21–28 kDa (42). The systemic (serum/plasma) levels usually range from 1.8 to 14 pg/ml in healthy individuals with no significant age-dependent differences (43, 44). During inflammation a more than 10^5^-fold increase can be observed, often correlating with disease severity (45). IL-6 is released predominantly by cells of mesenchymal origin, fibroblasts, muscle cells, keratinocytes, monocytes, and macrophages (46) but can also be released by endothelial cells. During acute inflammation the main transcription factors responsible for the increased IL-6 expression are NF-κB, CEBP-α, and AP-1 (47–49); these are activated by TNFα, IL-1β, the toll-receptor pathway, prostaglandins, and adipokines (50). IL-6 expression is also regulated by several miRNAs and RNases. Macrophages and monocytes appear to be the main sources of IL-6 during acute inflammation, while T cells seem to be a major contributor during chronic inflammation (51). However, IL-6 is also released in several physiological settings, e.g., during exercise when IL-6 released by muscle tissue can cause a 100-fold increase in systemic levels (52).

### The IL-6 Receptor—Classical versus Trans-Signaling and IL-6 Cluster Signaling

The IL-6 receptor exists both in a membrane-bound and a soluble form; it has only a short intracellular domain and relies on the ubiquitously expressed gp130 cell membrane protein for intracellular signal transduction (10). The membrane-bound receptor is only expressed by a limited number of cell types, including hepatocytes, neutrophils, naïve T cells, macrophages, and a subset of intestinal epithelial cells. Thus, in these cells, IL-6R signaling can be initiated through the membrane-associated complex of IL-6, IL-6R, and gp130. This is termed classical signaling and is often associated with tissue regeneration and anti-inflammatory properties (10).

**Figure 2:**
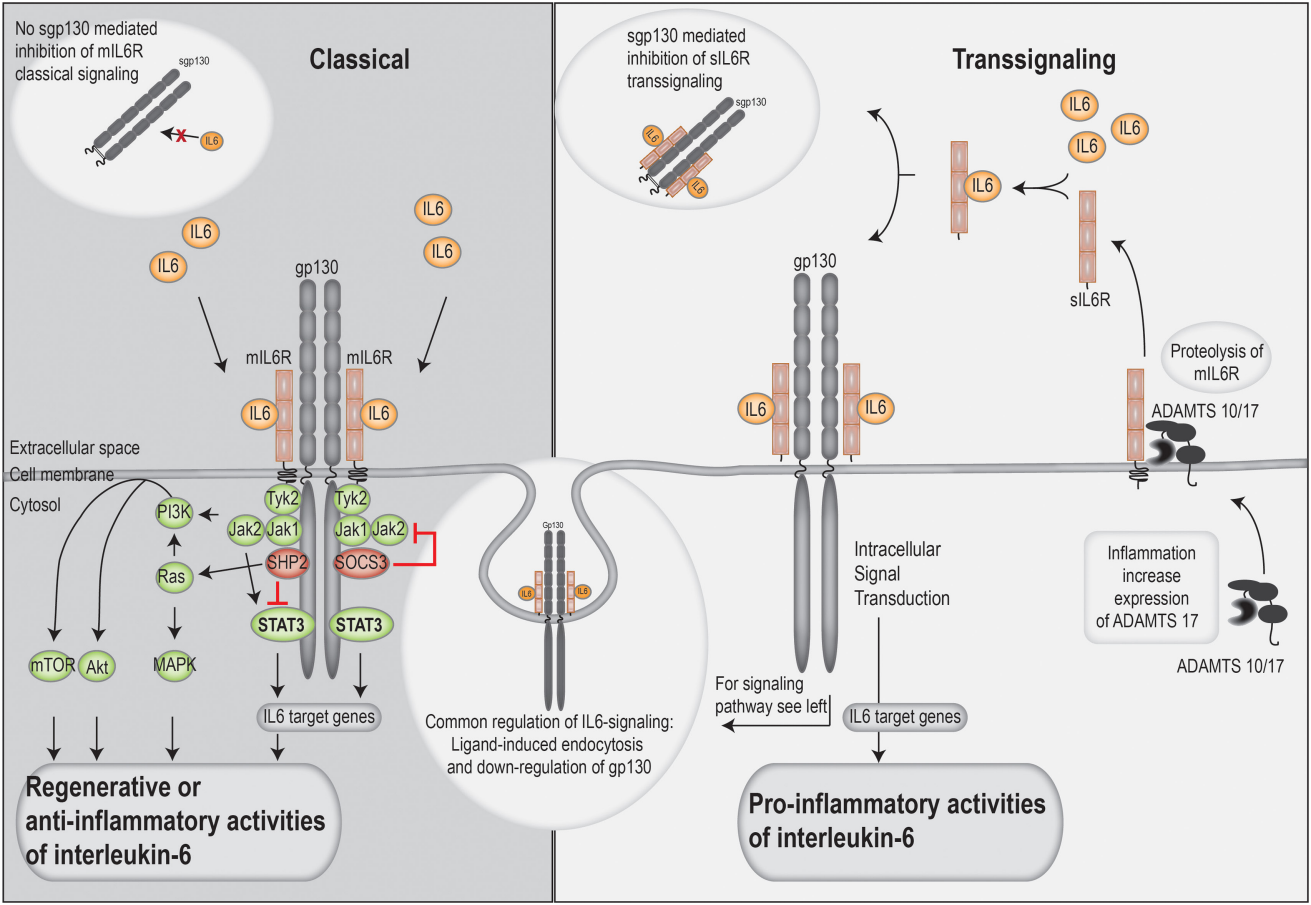
An overview of the classical and trans-signaling.

Most soluble cytokine receptors inactivate cytokines by interfering with membrane receptor binding, whereas the soluble IL-6R receptor in complex with IL-6 binds and activates gp130 on cells that do not express the membrane-bound IL-6R (mIL-6R). Thus, cells that do not express the IL-6R themselves can be IL-6 responsive; this mode of activation is called trans-signaling and seems important for many of the pro-inflammatory effects of IL-6 (Figure 2) (42).

Soluble IL-6R is mainly formed through cleavage of mIL-6R by a disintegrin and metalloprotease (ADAM) 10 and 17 proteases. The soluble receptor is constitutively released by the liver and by hematopoietic cells, but activation of ADAM17 during inflammation causes a rapid local increase in sIL-6R levels.

Dimeric gp130 is produced by alternative splicing and is secreted into serum in a soluble form; this dimer cannot bind IL-6 alone but only IL-6 in complex with sIL-6R. Classical IL-6 signaling will then remain intact because of the free IL-6 and unaffected membrane-bound IL-6R, whereas IL-6 trans-signaling is quenched due to the binding of the soluble IL-6/IL-6R dimeric complex. Under normal circumstances the sgp130 has a higher molar concentration than the sIL-6R (two times higher), and gp130 thereby acts as a physiological buffer against IL-6 trans-activation. Thus, IL-6 trans-signaling is regulated both by the release of the sIL-6R and the level of soluble gp130.

A third mode of IL-6 signaling termed IL-6 cluster signaling has recently been described (53). IL-6 is then loaded onto the IL-6 receptor as an intracellular process and thereafter expressed on the cell surface membrane of APC where the complex directly stimulates gp130 on T cells by direct cell–cell contact (53). So far, cluster signaling has only been described for the development of pathogenic Th17 cells in mice.

### The IL-6 Cytokine Family

The IL-6 family includes IL-11, IL-27, IL-31, leukemia inhibitory factor (LIF), OCM, ciliary neurotrophic factor (CNTF), CT-1, NP, CLC, CT-2, and humanin (Table 1). These proteins share structural resemblance with IL-6 and utilize gp130 or a gp130-like molecule for intracellular signal transduction (54). However, more ligand-specific receptor subunits are also used, including leukemia inhibitory factor receptor, OSMR, and IL-27RA. Some of these ligand-specific receptors have intracellular domains that can stimulate other signaling cascades than that of gp130. There is also cross-reactivity between the different IL-6 family cytokines and their receptors. Based on the different combinations of the utilized transmembrane proteins, the IL-6 cytokine family can be divided into different subgroups (Table 1) (55).

**Table 1:** The IL-6 cytokine family—an overview of the receptor ligands/cytokines, their receptor structure, and the different subgroups based on receptor complex.

Receptor ligands	Transmembrane signal transducer	Different receptor complexes
IL-6	Dimeric gp130	IL-6R + gp130
IL-11		IL-1R + gp130

LIF	LIFR/gp130	LIFR + gp130
CT-1		LIFR + CT-1R + gp130
OSM		LIFR + gp130

CNTF	LIFR/gp130/CNTFR	LIFR + CNTFR + gp130
CT-2		LIFR + CNTFR + gp130
CLC		LIFR + sCNTFR + gp130 or LIFR + CTNFR + CLR + gp130

IL-27	WSX-1/gp130	IL-27RA + gp130
Humanin		IL-27RA + CNTFR + gp130

IL-31	OSMR or gp130-like (Gpl)	OSMR + IL-31R (Gpl)
OSM		OSMR + gp130

*IL-6, interleukin-6; LIF, leukemia inhibitory factor; LIFR, LIF receptor; OSMR, OSM-receptor; CNTF, ciliary neurotrophic factor; CNTFR, CNTF receptor; sCNTFR, soluble CNTF receptor; CT-1R, CT-1 receptor; WSX-1 alias:IL-27RA*.

### Receptor-Initiated Intracellular Signal Transduction

Gp130 is non-covalently associated with the Janus kinases (JAKs) JAK1, JAK2, and TYK2. Following receptor ligation, the JAKs are auto-phosphorylated, and they also phosphorylate gp130. This phosphorylation provides docking sites for phosphorylation of STAT1, STAT3, and the tyrosine phosphatase SHP-2. After phosphorylation, STAT3 dimerizes and is translocated to the nucleus where it acts as a transcription factor (56). Furthermore, SHP-2 activates the RAS/RAF/MAPK/ERK pathway, whereas gp130 activation also leads to activation of the PI3k–AKT pathway together with the transcriptional regulator YAP1 (Figure 2). Most of the IL-6 effects seem to be STAT3-mediated. STAT3 is controlled by a negative feedback mechanism; it induces expression of SOCS proteins and activation of the SHP-2 phosphatase. SOCS3 then binds with high affinity to the same phosphorylated binding site on gp130 as JAK1/2 and thereby inhibits further intracellular signaling. Even though SOCS1 can also bind to the same site, this interaction is believed to be functionally less important *in vivo*. IL-6 signaling is also inhibited by internalization and degradation of the receptor complex, and internalization of gp130 prevents further signaling of the IL-6 cytokines.

### Experimental Tools for Examining Classical IL-6 Signaling and Trans-Signaling

The development of the two designer proteins sgp130FC and Hyper-IL-6 has made it possible to investigate the *in vitro* and *in vivo* effects of IL-6 classical and trans-signaling (42). The sgp130FC is constituted of two monomeric sgp130 molecules coupled with a human FC region. The affinity of sgp130FC to the IL-6/sIL-6 complex is 100- to 1,000-folds higher than naturally occurring sgp130 monomers, and it can abolish IL-6 trans-signaling without affecting the classical IL-6 signaling. The designer cytokines Hyper-IL-6 consists of IL-6 that is directly linked with the IL-6 receptor. Hyper IL-6 binds and activates gp130 on cells not expressing mIL-6 receptor. The use of these molecules in several mouse models has given valuable insight into IL-6 biology, e.g., antigen-induced arthritis, inflammatory bowel disease, colitis associated cancer, pancreatitis induced acute lung injury, and hepatocellular carcinoma model (57–61).

## IL-6, the Acute Phase Response and the Risk of GVHD

The acute phase response represents a physiological increase in the levels of certain serum proteins due to increased production and release especially by the liver, and this includes C-reactive protein (CRP), serum amyloid P, ferritin, mannose binding protein, and fibrinogen (62). IL-6 is the main driver of the response, but other cytokines (IL-1, IL-8/CXCL8, and TNFα) also contribute (62). The levels of several acute phase proteins (e.g., CRP) is strongly correlated with IL-6 levels, and the IL-6 levels are again often correlated with the extent of tissue damage. In some malignancies there is evidence of chronic inflammation with a persisting acute phase response; but this response may also be due to constitutive IL-6 release by malignant cells (63, 64).

Both IL-6 and CRP levels are elevated in most patients prior to ASCT, and this may be due to chronic fungal or bacterial infections, GI barrier break, or residual malignant disease (65, 66). The impact of pretransplant CRP levels after allotransplantation has been investigated in several studies (6, 65–71). Elevated CRP levels seem then to independently entail a higher transplant-related morality without increasing the risk of acute or chronic GVHD. The effect of both CRP and IL-6 levels has only been investigated in two studies (6, 66). Although CRP and IL-6 serum levels are highly correlated, CRP seems to independently influence the TRM, while the pretransplant IL-6 level does not seem to have a similar impact on TRM or GVHD. A possible explanation for this could be that the CRP levels are influenced by several comorbidities and not only IL-6.

## IL-6, Effects on Immunocompetent Cells

Interleukin-6 has both direct and indirect effects on immunocompetent cells involved in the development of GVHD. Several of these effects are summarized in Table 2. First, IL-6 is essential for maturation, proliferation, differentiation, and maintenance of B cells/plasma cells and several proinflammatory T-cell subsets. IL-6 seems to enhance the development of pro-inflammatory Th17 and Th2 cell and to inhibit the development T regulatory cells. Second, IL-6-signaling is crucial for trafficking of immune cells to inflamed tissues and lymphoid organs. This is caused both by altered expression of adhesion molecules by endothelial cells and by expression of their ligands by immunocompetent cells. Third, IL-6 has important functions in GVHD target organs, and there may therefore be a risk of combined injury during GVHD (e.g., GVHD-induced immune-mediated damage, pharmacological toxicity, and IL-6 inhibition). Finally, IL-6 together with TNF-α released from macrophages has been reported to directly/independently contribute to tissue damage in GVHD (40).

**Table 2:** Important effects of interleukin-6 (IL-6) on immunocompetent cells involved graft-versus-host disease (GVHD) and on GVHD target organs.

IL-6 effects on immunocompetent cells
**T cells:** Membrane-bound IL-6R mainly expressed on naïve and memory T cells (60, 72).
**Naïve T cells:** IL-6 causes STAT3 activation, resulting in SOCS1 expression and thereby inhibited Th1 polarization (73).
**Th1 cells:** Suppressed Th1 development (73).
**Th2 cells:** Enhanced Th2/polarization/development through STAT3 dependent c-maf expression and STAT3 independent NFAT expression (73).
**Th17 cells:** Enhanced Th17 development through IL-6- and IL-21-induced STAT3 activation followed by increased RORγT expression (56, 73–76). Differentiation from naïve T cells to Th17 relies largely on classical IL-6 signaling, whereas maintenance of Th17 cells depends on trans-signaling (60). STAT3 activation in naïve T cells by mIL-6R/IL-6 complex on the dendritic cells (cluster signaling) has been shown to be important for the development of pathogenic Th17 cells (53).
**Th22 cells:** The development depends on combined effects of IL-6, TNFα, IL-1β, and the aryl hydrocarbon receptor (77).
**Tregs:** Suppressed Treg development, inhibition of FOXP3 expression. Indirect effects of IL-6 may increase Treg development through increased release of anti-inflammatory cytokines (78).
**Tfh:** IL-6 seems to commit T cells to Thf differentiation (79, 80).
**Dendritic cells and monocytes**
**Dendritic cells:** Inhibits differentiation of dendritic cell from bone marrow progenitors, and decreased responsiveness of dendritic cells. STAT3 activation seems important for these effects (81, 82).
**Monocytes:** Increased M-CSF expression favors differentiation into macrophages rather than dendritic cells (83).
**B cells:** A key factor for regulation of B cell survival and maturation through both direct as well as indirect effects by stimulation of Tfh development (79, 80). Supports development into long-lived plasma cells (84).
**Mesenchymal stem cells (MSCs):** MSCs have unique immunomodulatory effects and have therefore been used in the treatment of acute and chronic GVHD. MSCs suppress Th1 and Th17 cells and induce Treg expansion through the release of multiple cytokines, including IL-6 and the IL-6 family cytokine LIF (85). Pro-inflammatory signals upregulate the constitutive IL-6 release by MSCs (86), and autocrine IL-6 stimulation will thereafter induce the release of immunosuppressive prostaglandin E2 by the MSCs (87). Furthermore, LIF secretion by MSCs inhibits T-cell proliferation in mixed lymphocyte reaction and seems to enhance the generation of Tregs (88). Autocrine IL-6 stimulation seems important for their survival, maintenance of stemness, and regulation of proliferation; ERK1/2 seems important for these effects (89, 90). MSC/T cell cross talk seems to increase local IL-6 levels (91).
**Effects of IL-6 on leukocyte migration during local inflammation**
Enhances recruitment of primed T cells to inflamed tissue and entry of naïve T cells to lymphoid organs (54). Fever alone increases leukocyte extravasation through gp130 dependent mechanisms (92). IL-6 trans-signaling as well as signaling initiated by other IL-6 family members increase l-selectin expression by T cells through ERK1/2 activation and increase their extravasation (93, 94). IL-6 trans-signaling increases vascular expression of both adhesion the molecules (e.g., ICAM-1, VCAM-1, CD62E, and release of chemoattractant (CCL2, CXCL10, CCL4, CCL5, CCL11, and CCL17) (95).

## IL-6 a Regulator of Stem Cells and Tissue Regeneration

Interleukin-6 is important for the regulation of stem cells and tissue regeneration in several organs. This has been best demonstrated for hematopoiesis, liver cells, GI mucosa, and muscle cells. A complete overview of these effects is beyond the scope of this article. A brief overview of the major effects of IL-6 in these different organ systems is given in Table 3. Impaired IL-6 function in these organs is associated with reduced regeneration after injury, and IL-6 dysregulation during chronic inflammation can contribute to organ dysfunction.

**Table 3:** Important effects of interleukin-6 (IL-6) in target organs of graft-versus-host disease (GVHD) and on metabolism.

IL-6 and the target organs of GVHD
**Liver:** IL-6 together with other cytokines is important for liver regeneration; the prolonged effect of trans-stimulation seems more important than classical signaling (96, 97). Clinical IL-6 inhibition can cause increased liver transaminases (98), but it is not known whether IL-6 targeting, potentially liver-toxic drugs and concomitant immune-mediated injury due to GVHD, will increase the risk of severe liver toxicity in allotransplant recipients.
**Gastrointestinal mucosa:** IL-6/STAT3 is important for regeneration of intestinal epithelium, maintain barrier integrity, ensure adequate secretion of antimicrobial peptide, support proliferation, and facilitate migration of intraepithelial lymphocytes (99–101). Inhibition of IL-6 signaling (especially trans-activation) suppresses colitis (59, 102). Clinical use of IL-6 inhibition is associated with increased risk of bowel perforation (103).
**IL-6 and the effects on metabolism**
**Cachexia:** High IL-6 levels are often observed in patients with cachexia. Although some studies have shown that IL-6 blockade can attenuate anemia and muscle loss in cancer patients, the role of IL-6 in the metabolic changes during cachexia is still unclear.
**Glucose tolerance:** Obesity/glucose intolerance is associated with low-grade chronic inflammation and high baseline IL-6 levels (104). Insulin treatment reduces IL-6 levels (105, 106). IL-6 secreted by adipocytes seems to favor the development of anti-inflammatory M2 (107). Inhibition of the mIL-6R in the liver is associated with reduced glucose tolerance and dyslipidemia (108).
**Muscle cells:** IL-6 is important in regeneration of muscle after injury through a direct effect on muscle stem cell-IL-6 (109). IL-6 is also actively secreted by muscle cells and has important endocrine and metabolic effects.

## The Role of IL-6 in GVHD—Lessons from Animal Models

The role of IL-6 in acute and chronic GVHD has been investigated in several mouse models. Givon et al. (110) examined the effect of IL-6 on bone marrow reconstitution after syngeneic, semi-allogenic, and ASCT and found that posttransplant treatment with subcutaneous recombinant IL-6 significantly supported white blood count reconstitution and improved survival in syngeneic as well as allogenic transplantation. This effect was only significant for animals transplanted with a low stem cell dose. In contrast, mice receiving IL-6 showed increases in both the severity of and mortality from GVHD.

Chen et al. (111) observed increased systemic IL-6 and IL-6R levels early after both syngeneic and allogeneic transplantation. These levels returned to baseline over time in the syngeneic group, whereas IL-6 levels remained high in mice developing GVHD. Both IL-6 and IL-6R expression increased in the liver and colon (but were stable in the spleen), and the highest IL-6R mRNA levels were observed in these two organs. Selective IL-6 knockout in neither recipient nor donor cells was sufficient to protect from GVHD. However, GVHD treatment with anti-IL-6 resulted in significantly lower weight loss, less histopathological evidence of damage to colon, liver, and lungs, and significantly increased Treg levels in the spleen. The increased Treg levels were not dependent on an intact thymus; rather the IL-6 blockade increased peripheral generation of Treg cells and reduced the levels of Th1 and Th17 cells. Similar results were shown by Noguchi et al. (112); treatment with an anti-IL-6 antibody reduced liver enzyme levels as well as the occurrence of organ failure and was associated with reduced infiltration of Th1 and Th17 cells, increased Treg cells, and improved survival.

Tawara et al. (113) investigated the effect of IL-6 derived from donor T cells. In contrast to the findings by Chen et al., selective IL-6 knockout in donor/graft T cells was associated with less severe GVHD and prolonged survival. Pretransplant anti-IL-6 treatment also significantly improved survival and clinical as well as histopathological severity of GVHD, but the Treg levels were not altered. Importantly, the GVL effect was maintained despite the reduction in GVHD. The systemic cytokine levels and the levels of circulating cells were not altered by the treatment, and selective ablation of IL-6 in recipient bone marrow cells did not reduce the occurrence or severity of GVHD.

Organ-specific effects of IL-6 in GVHD have been investigated in two different mouse models. Varelias et al. (36) examined the role of IL-6 in idiopathic pulmonary syndrome (IPS) after ASCT and demonstrated that local secretion of IL-6 induced Th17 cell differentiation that was necessary for disease development. IPS development could be prevented by IL-17 knockout or by the use of neutralizing antibodies against IL-17. In a second study, Le Huu et al. (114) investigated the role of IL-6 in a sclerodermous cGVHD model and observed that IL-6 levels increased during disease progression. Treatment with IL-6-neutralizing antibodies prior to manifestations of scleroderma significantly decreased disease severity, while no reduction was observed when IL-6 neutralization was started after the onset of cGVHD. Treatment with anti-IL-6 was associated with a significant increase in the number of splenic Treg cells, whereas the expression of IFN-γ, TNFα, IL-6, IL-18, TGF-β1, CCL2, CCL3, and CCL5 in affected skin was significantly reduced.

## Experimental Studies Suggest a Role of JAK2–STAT3 Activation in Allorecognition and GVHD Development

The role of STAT3 in the regulation of activation and differentiation of Tregs and Th17 cells after allotransplantation has been investigated in several mouse models (i.e., STAT3-knockout mice) and *in vitro* models. Emerging evidence also suggests that activation of STATs in B cells is also important for GVHD (115). A complete review of this scientific field is beyond the scope of this article, but an overview of important observations is given in Table 4 (116–120). In summary, these reports suggest that targeting of IL-6/JAK2/STAT3 signaling can be effective against the development of GVHD. Treatment can be given either by graft engineering or as posttransplant therapy, the function of several immunocompetent cells will then be affected, and reduced GVHD severity as well as improved survival has been observed. Based on these findings, treatment with the JAK1/2 inhibitor ruxolitinib has been tested in patients with manifest chronic and aGVHD, and it was reported to improve GVHD and reduce serum cytokine levels (121, 122). Patients included in these two studies are heterogeneous with respect to GVHD manifestations, previously GVHD prophylaxis and treatment. Further well-designed studies are definitely needed to determine the efficacy and toxicity of JAK2/STAT3 blockade in allotransplant recipients.

**Table 4:** The role of STAT3 in regulation of activation and differentiation of immune cells in allogeneic stem cell transplantation; important observations in animal studies and experimental *in vitro* studies.

**Pretransplant intervention:** Pretransplant inhibition of STAT3 in graft T cells through upstream pharmacological JAK2 inhibition reduced graft-versus-host disease (GVHD) mortality and increased the levels of Tregs (116).
**GVHD mortality:** STAT3 ablation reduces posttransplant mortality as well as the severity of both acute and chronic GVHD, especially GVHD severity in the skin and colon (119, 120).
**Tolerance induction:** JAK2 inhibition seems to be more effective than interleukin-6 (IL-6) neutralization for induction of immunological tolerance (117, 118).
**T cell proliferation:** Proliferating alloreactive T cells expressed higher levels of phosphorylated STAT3 compared to proliferating T cells in syngeneic transplantations (116).
**Dendritic cells:** Pharmacological inhibition of STAT3 phosphorylation by IL-6 neutralizing antibodies or JAK2 inhibitors does not inhibit dendritic cells (117).
**Regulatory T cells:** Equal numbers of Treg cells are observed in both STAT3-wt and knockout mice after syngenic transplantation, while after allotransplantation mice STAT3 expression is associated with a lower number of Tregs cells (119).
The STAT3 effects differ between nTregs and iTregs; nTregs with impaired STAT3 function do not prevent GVHD, whereas STAT3 knockdown in iTreg prevents GVHD (119).

## IL-6 in ASCT—Clinical Data

### aGVHD and IL-6 Polymorphism

Specific single nucleotide polymorphisms (SNPs) in the IL-6R and the IL-6 gene are associated with higher serum levels of IL-6 and sIL-6R. Especially the SNP 174 G < C (rs1800795) in the IL-6 promoter region affects the transcription and secretion of IL-6 and has been associated with several autoimmune disorders (123, 124). A summary of the 10 studies that have examined the role of this specific SNP in acute and chronic GVHD is presented in Table 5; of note, only one of those studies examined effects of SNPs both in the IL-6 and IL-6R gene (125–137). A meta-analysis of seven studies published during the period 2001–2012 was performed by Choi et al. (138); they concluded that patients who received grafts from donors either hetero- or homozygous for the IL-6 G allele had significantly increased risk of developing aGVHD grade I–IV (odds ratio 3.30) or grade II–IV (odds ratio 1.73). To date, the largest study investigating the role of IL-6 polymorphisms in GVHD is that of Chien et al. (128), which included 1,298 patients. They investigated IL-6 SNP polymorphisms together with 14 other candidate genes, and their unadjusted and adjusted analyses of patients with unrelated donors showed that the donor genotype rs1800795 was associated with a 20–50% increased risk of grade II–IV aGVHD.

**Table 5:** Summary of studies investigating the influence of different SNPs in the IL-6 and IL-6R on outcome after allogenic stem cell transplantation.

Reference	Year	Patients	Donor type	Stem cell source		aGVHD		cGVHD	Survival
				PB	BM	Genotype	Risk		
**IL-6 rs1800795 or rs1800797**									
Alam et al. (133)	2015	268	MRD 184	184	NR	D	Increased	NR	No effect
Chien et al. (128)	2012	1,298	Related 612	377	921	D	Increased	NR	NR
			Unrelated 686						
Ambruzova et al. (125)	2008	56	Sibling	NR	NR	D	Increased	No	Decreased
Ambruzova et al. (126)	2009	166	121 related	144	22	R		No	Borderline decreased
			45 unrelated						
Karabon et al. (134)	2005	93	Sibling	NR	NR	D/R	Increased	NR	No
Laguila Visentainer et al. (131)	2005	118	Sibling	36	82		No effect	Increased	No effect
Mullighan et al. (137)	2004	160	154 sibling	100	60	D	Increased	No	No effect
Lin et al. (132)	2003	993	Sibling				No effect	NR	NR
Rocha et al. (136)	2002	107	Sibling		107		No effect	No effect	No effect
Socié et al. (135)	2001	100	Sibling				No effect	Increased risk	NR
Cavet et al. (127)	2001	80	Sibling	80		R	Tendency higher grade	R or D increased	NR

**IL-6R polymorphism, rs4845617**									
Kim et al. (129, 130)	2012, 2014	394	MRD 288	276	118	Increased risk chronic eye GVHD			
			MMD19			Recipient genotype associated with increased NRM			
			MUD84						

*BM, bone marrow; D, donor; MMD, mismatched related donor; MRD, matched related donor; MUD, matched unrelated donor; NR, not reported; NRM, non-relapse mortality; PB, peripheral blood stem cell collection; R, recipient; aGVHD, acute GVHD; SNPs, single nucleotide polymorphisms; GVHD, graft-versus-host disease*.

Kim et al. (129, 130) investigated the effects of 259 different SNPs on outcome after ASCT. While they did not observe any effect of SNPs in the IL-6 gene, patients with an SNP in the IL-6R gene (rs4845617) showed decreased relapse-free survival. In a new study of the same patients, univariate analysis identified several SNPs in the IL-6R (rs2229238, rs4072391, rs4379670, and rs7514452) that were associated with an increased risk of aGVHD, but they could not predict aGVHD in a multivariate analysis. The SNP (rs4845617) was predictive of cGVHD of the eyes and could be included in a model to predict GVHD.

The IL-6 receptor Asp358Ala SNP (rs2228145) increases the proteolytic conversion rates by ADAM proteases, and this leads to higher soluble IL-6R levels; it is also associated with a lower baseline CRP and decreased incidence of autoimmune disorders (139). To the best of our knowledge, the effect of this specific SNP on the risk of complications after allotransplantation has not been investigated.

### IL-6 Level and Clinical Outcome

Pretransplant IL-6 levels in allotransplant recipients show a wide variation and a strong correlation with pretransplant CRP levels but do not seem to predict survival, risk of GVHD, or non-relapse mortality after transplantation (6). This study included only patients with related donors (mainly matched sibling donors) transplanted in complete remission (all except 1 of the 99 patients), and the pretransplant systemic levels of all IL-6 family members were studied. Only pretransplant IL-31 influenced late transplant-related mortality. However, there was a significant association between pretransplant IL-6 and early postconditioning weight gain (i.e., fluid retention), and this fluid retention in turn was a risk factor for aGVHD, transplant-related mortality, and overall survival. The authors concluded that pretransplant CRP, pretransplant IL-31, and early posttransplant fluid retention were independent risk factors for transplant-related mortality and survival after allotransplantation. The strong association between pretransplant IL-6 levels and vascular permeability/fluid retention suggests that effects of IL-6 on endothelial cells may contribute to a high-risk phenotype.

After onset of chemotherapy, IL-6 levels rise significantly and reach a maximum during the two first weeks before returning to baseline in most patients (140, 141). Increased systemic IL-6 levels are also observed during febrile illnesses and toxic complications in patients with hematological malignancies. Both after intensive chemotherapy and allotransplantation, the levels are strongly associated with the CRP levels and are accompanied by a decrease in sIL-6R levels (142–144). Conflicting results have been reported about IL-6 levels in aGVHD; some have reported high levels prior to aGVHD (36, 145, 146), whereas others have not been able to verify this (147–149). Increased IL-6 levels have also been associated with severe pulmonary toxicity and with reduced survival after autologous stem cell transplantation (140, 150).

### IL-6 Blockade in Prevention of GVHD

Only two drugs inhibiting IL-6 or the IL-6R are currently approved for clinical use; tocilizumab is used in rheumatoid arthritis and systemic sclerosis, whereas siltuximab is approved for treatment of multicentric Castleman’s disease (Table 6). Tocilizumab is a humanized monoclonal antibody that binds to both membrane-bound and soluble IL-6R, while siltuximab is a chimeric monoclonal antibody binding circulating IL-6 (151). Tocilizumab is generally well tolerated in patients with rheumatoid arthritis; the most commonly reported side effects being dyslipidemia (21–25%), increased liver transaminases (5–6%), and transient decreases in neutrophil counts (152–154). IL-6 blockade inhibits the acute phase response and may thereby mask signs of acute severe infections. Although some studies in rheumatoid arthritis have shown a higher rate of infections in patients treated with tocilizumab (153, 154), this could not be confirmed in a large multicenter study (152). Several other IL-6 inhibitory small molecules and monoclonal antibodies are currently in clinical trials both for autoimmune disorders and different types of cancer (151).

**Table 6:** A comparison of the two IL-6 blocking monoclonal antibodies approved for clinical use.

Characteristics	Tocilicumab	Siltuximab
Specificity	Anti IL-6R (membrane and soluble)	Anti IL-6
Antibody structure	Humanized	Chimeric (mouse/human)
Administration	Subcutaneous or intravenous	Intravenous
Approved indications	Rheumatoid arthritis	HIV-negative muticentric Castleman’s disease
	Systemic juvenile idiopathic arthritis	
Example of reported off-label use	Cytokine release syndrome after CAR-T therapy	
	Giant cell arteritis	
	Graft-versus-host disease	
	Adult Still’s disease	

IL-6 blockade as prophylaxis against GVHD has been investigated in only one study (155), where a single dose of tocilizumab 8 mg/kg (maximum dose 800 mg) was added to standard GVHD prophylaxis with cyclosporine A and methotrexate in 48 patients that underwent T-cell replete allotransplantation with myeloablative or reduced intensity conditioning regimes. The incidence of grade III–IV aGVHD and the non-relapse mortality were only 4%. The incidence of severe aGVHD was also low in patients receiving myeloablative conditioning based on total body irradiation; a treatment protocol usually associated with a high risk of GVHD. The addition of tocilizumab appeared generally safe with no increase in graft rejections, time to neutrophil regeneration, chimerism at day + 40 posttransplant, or early relapse compared to historical controls. Only three patients experienced severe liver toxicity during the first month after transplantation.

To our knowledge, there is only one ongoing trial that investigates the role of adding IL-6 blockade to standard prophylactic GVHD-therapy (NCT02206035). In this open label phase II trial, tocilizumab is given in addition to tacrolimus/methotrexate and compared with a contemporary control cohort. The primary endpoint is grade II–IV GVHD during the first 180 days posttransplant. In a second trial, the rate of cytokines release syndrome with and without IL-6 blockade with tocilizumab will be investigated in a small number of patients receiving haploidentical transplantation with posttransplantation cyclophosphamide (NCT02057770).

### IL-6 Blockade in the Treatment of GVHD

In addition to a few case reports (156–158) and conference abstracts (159) only two published case series report the effects of tocilizumab in the treatment of acute steroid-refractory aGVHD (Table 7) (160, 161). Drobyski et al. (161) described the effects in eight patients (two acute and two chronic GVHD) where tocilizumab was administered once every 3–4 weeks. Five patients had grade IV GVHD (four gut and one skin involvement), while one patient had grade II gut, and the last patient had grade III liver affection. One of these patients died early after tocilizumab administration and was not evaluable, one patient did not respond, three patients were classified as partial responders, and two patients were considered complete responders. In one of the patients with partial response, the treatment was discontinued because one could not exclude the possibility that tocilizumab worsened preexisting hyperbilirubinemia. A transient increase in liver transaminases was also observed in several patients. The authors concluded that infections were responsible for the major adverse events associated with tocilizumab administration, with a total of 13 documented infections.

**Table 7:** Summary of studies with IL-6 receptor (tocilizumab) or JAK2 (ruxolitinib) blockade in treatment of chronic and acute steroid-refractory GVHD.

Reference	Patients (*n*)	aGVHD (*n*)	cGVHD (*n*)	Organ involvement (patients)			Response aGVHD	Response cGVHD
				Skin	Gastrointestinal	Liver		
**Tocilizumab**								
Drobyski et al. (161)	8	6	2	Grade II: 3	Grade II: 1	Grade III: 1	CR 2, PR 2, NR1, NR 1	1 Stabilization 1 PR
				Grade IV: 1	Grade III: 4			
Roddy et al. (160)	9	9	0	Grade II: 1	Grade I: 1	Grade I: 1	CR: 2, CR in single organ system 2	N/A
					Grade II: 2	Grade II: 2		
					Grade III: 2	Grade III: 1		
					Grade IV: 4	Grade IV: 2		
Ganetsky et al. (159)	5	5		All patients Glücksberg grade IV			CR 5	N/A

**Ruxolitinib**								
Zeiser et al. (122)	95	54	41	All patients with aGVHD grade III/IV, not otherwise specified			ORR 44, CR 25	35
Spoerl et al. (121)	6	4	2	Grade III: 2, Grade IV: 1	Grade IV: 2	Grade III: 1	CR 1, PR 3	2

*CR, complete response; PR, partial response; NR, non-response; ORR, overall response rate; N/A, not applicable; GVHD, graft-versus-host disease; aGVHD, acute GVHD*.

Roddy et al. (160) reported the effect of tocilizumab in patients with steroid-refractory aGVHD; seven patients with grade IV and two with grade III aGVHD. Tocilizumab was administered in a similar manner as in the previously reported study. Five patients were classified as non-responders, two patients were classified as complete responder, and two patients had mixed response with persistence of severe aGVHD in one organ but resolved disease in other organs. In this study, four patients suffered from infectious episodes with two deaths being reported, but no liver toxicity was observed. Only one ongoing study for treatment of steroid-refractory aGVHD has been registered (https://clinicaltrials.gov, NCT01475162), but this study was ended because the risks of toxicity seemed to outweigh the potential benefits.

## Concluding Remarks

Graft-versus-host disease is a complex, multi-systemic disease in which local and systemic factors play a role in orchestrating inflammation, tissue repair, and regeneration. The impact of SNPs in the IL-6 gene on GVHD risk indicates that IL-6 plays a role during early GVHD development. Blockade of the pro-inflammatory effects of IL-6 seems to be a possible therapeutic strategy since IL-6 and STAT3 activation are closely linked to the development of both Th17 and Treg cells, and since early STAT3 phosphorylation posttransplant seems to precede development of GVHD (118). However, currently available clinical data indicate that IL-6 blockade is most effective when used as GVHD prophylaxis, whereas manifest GVHD might be less susceptible to IL-6 blockade. An unselective systemic blockade of IL-6 is probably not without caveats, given the physiological importance of IL-6 in tissue regeneration and homeostasis in both the liver and GI tract, two organs commonly affected both in acute and chronic GVHD.

Most of the pro-inflammatory effects of IL-6 seem to be caused by trans-signaling. A fusion protein consisting of the immunoglobulin Fc region and gp130 can be used for specific blocking of trans-signaling (56); this protein binds IL-6 in complex with sIL-6R but cannot bind free IL-6. Treatment with this selective inhibitor inhibits inflammation and preserves the regenerative effects of IL-6 in mouse models of a wide variety of diseases, e.g., ulcerative colitis, rheumatoid arthritis, and acute pancreatitis-associated lung injury (56). All experimental models of GVHD explored so far have utilized either genetic or pharmacological blocking of IL-6, and little is therefore known about the relative contributions of trans- versus cis-/classical IL-6 signaling in these settings. Further models should therefore address the use of selective blockade of IL-6 trans-signaling after ASCT.

## Author Contributions

TT, EE, AT, and ØB contributed equally to this work.

## Conflict of Interest Statement

The authors declare that the research was conducted in the absence of any commercial or financial relationships that could be construed as a potential conflict of interest.

## Abbreviations

ADAM, a disintegrin and metalloproteinase; AP-1, activator protein 1; ASCT, allogeneic stem cell transplantation; C/EBP-α, CCAAT/enhancer-binding protein alpha; CCL, chemokine (C–C motif) ligand 5; CNTF, ciliary neurotrophic factor; CRP, C-reactive proteins; CT-1, cardiotrophin-1; CT-2, cardiotrophin-2; DAMPs, damage-associated molecular patterns; APC, antigen-presenting cells; MHC, major histocompatibility complex; EGF, epidermal growth factor; ERK, extracellular signal-regulated kinases; FOXP3, forkhead box P3; GATA-3, globin transcription factor 3; GM-CSF, granulocyte–macrophage colony-stimulating factor; gp130, glycoprotein 130; GPL, gp130-like; GVHD, graft-versus-host disease; IBMTR, Center for International Blood & Marrow Transplant Research; ICAM-1, intercellular adhesion molecules; IFN-γ, interferon gamma; IL, interleukin; IL-6R, IL-6 receptor alpha; JAK, Janus kinase; LIF, leukemia inhibitory factor; LIFR, leukemia inhibitory factor receptor; MAPK, mitogen-activated protein kinase; MSCs, mesenchymal stem cells; NFAT, nuclear factor of activated T cells; NF-κB, nuclear factor kappa-light-chain-enhancer of activated B cells; NP, neuropoetin; OCM, oncostatin M; OSMR, oncostatin M receptor; PAMPs, pathogen-associated molecular patterns; RAF, rapidly accelerated fibrosarcoma; RAS, rat sarcoma; RORγT, RAR-related orphan receptor gamma; sIL-6R, soluble IL-6R; SNP, single nucleotide polymorphism; SOCS, suppressor of cytokine signaling; STAT, signal transducer and activator of transcription; TCR, T-cell receptor; Tfh, follicular B helper T cells; TGF, transforming growth factor; Th, T helper cells; TLR, toll-like receptor; TNFα, tumor necrosis factor alpha; Tregs, regulatory T cells; TYK, tyrosine kinase; VCAM-1, vascular cell adhesion protein 1; YAP1, yes-associated protein 1.
